# Supplementary material for: Active site specificity profiling datasets of matrix metalloproteinases (MMPs) 1, 2, 3, 7, 8, 9, 12, 13 and 14
Source: Data Brief. 2016 Feb 22;7:299–310. doi: 10.1016/j.dib.2016.02.036 (PMC4777984; doi:10.1016/j.dib.2016.02.036)
Supplement: Supplementary file 10 — Supplementary material [file mmc10.zip › WebPICS_hMMP14_G_1%/subsite_coop.html]

 

PICS results


- # About
- # Manual
- # Analysis
- # Results

- Redraw
- Seqlogo
- Dependency
- Coop

**Potential subsite cooperativity analysis for hMMP14\_G\_1%**   
Minimum difference for subsite dependency was set to +- 10 percentage-points  
Subsite dependency was checked for all positional occurences above 1 x natural abundance  
**Subsite dependency check is restricted to P3 - P3'**

|  |  |  |  |
| --- | --- | --- | --- |
| Fixed residue | Affected residue(s) | Change (percentage-points) | Vice-Versa change |
| P3\_A | P2\_A | -11.7 | -12.8 |
| P3\_A | P2prime\_L | 15.3 | 17.3 |
| P3\_C | P1\_H | 28.1 | 15.4 |
| P3\_C | P1prime\_C | 29.1 | 19.4 |
| P3\_C | P3prime\_M | 13.4 | 11.5 |
| P3\_C | P3prime\_Q | 44.4 | 22.2 |
| P3\_H | P1prime\_M | 33.0 | 11.0 |
| P3\_H | P2prime\_F | 33.9 | 13.1 |
| P3\_I | P1\_N | 36.9 | 21.1 |
| P3\_I | P2prime\_N | 26.5 | 42.5 |
| P3\_P | P1\_A | 17.8 | 14.6 |
| P3\_P | P1prime\_V | 11.5 | 20.6 |
| P3\_P | P2\_K | 16.5 | 22.3 |
| P3\_V | P2\_S | 12.8 | 17.3 |
| P3\_V | P2prime\_K | 23.7 | 12.3 |
| P3\_V | P3prime\_K | 18.3 | 20.6 |
| P2\_A | P3prime\_K | 14.5 | 18.7 |
| P2\_A | P3prime\_L | -11.4 | -11.4 |
| P2\_C | P3prime\_S | 15.6 | 10.1 |
| P2\_K | P1\_A | 29.5 | 17.9 |
| P2\_N | P1\_W | 10.6 | 42.5 |
| P2\_N | P2prime\_C | 10.6 | 42.5 |
| P2\_Q | P1\_Y | 23.1 | 28.1 |
| P2\_Q | P2prime\_L | 31.4 | 11.5 |
| P2\_Q | P3prime\_Q | 21.7 | 19.8 |
| P2\_R | P1prime\_F | 11.5 | 12.6 |
| P2\_S | P1\_S | 24.2 | 21.0 |
| P2\_Y | P1prime\_F | 19.8 | 14.4 |
| P2\_Y | P2prime\_F | 31.4 | 19.3 |
| P1\_A | P2prime\_K | 18.0 | 11.4 |
| P1\_C | P2prime\_M | 13.4 | 11.5 |
| P1\_G | P3prime\_V | 14.6 | 22.2 |
| P1\_H | P2prime\_N | 13.5 | 14.8 |
| P1\_H | P3prime\_K | 34.2 | 15.6 |
| P1\_P | P2prime\_M | 14.9 | 23.4 |
| P1\_Y | P3prime\_M | 30.0 | 38.7 |
| P1\_Y | P3prime\_Q | 27.7 | 20.8 |
| P1prime\_C | P3prime\_Q | 16.6 | 12.5 |
| P1prime\_F | P2prime\_F | 12.1 | 10.2 |
| P1prime\_F | P2prime\_R | 20.3 | 14.8 |
| P1prime\_F | P3prime\_N | 21.7 | 19.8 |
| P1prime\_I | P2prime\_L | 19.2 | 23.1 |
| P1prime\_I | P3prime\_A | 10.5 | 15.1 |
| P1prime\_M | P2prime\_F | 13.9 | 16.1 |
| P1prime\_M | P3prime\_T | 19.2 | 18.0 |
| P1prime\_Y | P3prime\_N | 23.0 | 13.4 |
| P2prime\_N | P3prime\_V | 28.3 | 11.3 |
| P2prime\_R | P3prime\_N | 14.4 | 18.0 |
| P2prime\_V | P3prime\_L | 20.7 | 11.4 |
| P2prime\_V | P3prime\_S | 16.9 | 20.6 |

  
**PICS analysis of protease: hMMP14\_G\_1%**  
213 cleavage sites analyzed  
PICS library made with (T)rypsin, (G)luC or (C)hymotrypsin:   
Cutoff for graphic display: 2 x natural abundance  

|  |  |
| --- | --- |
| Positional occurences  (table for total and relative (in %) values) | Occurences relative to natural abundance (table) |
|  |  |
